# Comparison of Partial Nail Avulsion With or Without Phenolization in the Management of Ingrown Toenails

**DOI:** 10.7759/cureus.83837

**Published:** 2025-05-10

**Authors:** Zaubaria Shehar Bano, Farooq Ahmad Rana, Aiza Irfan, Adan Tariq, Muhammad Saud Iqbal

**Affiliations:** 1 Surgery, Heartlands Hospital, University Hospitals Birmingham NHS Foundation Trust, Birmingham, GBR; 2 Surgery, Jinnah Hospital, Lahore, PAK; 3 General Surgery, Jinnah Hospital, Lahore, PAK; 4 Cardiac Surgery, Punjab Institute of Cardiology, Lahore, PAK; 5 General Surgery, Jinnah Burn and Reconstructive Surgery Centre, Lahore, PAK; 6 General Surgery, Allama Iqbal Medical College (AIMC), Lahore, PAK

**Keywords:** chemical matricectomy, ingrown toenail, ingrown toenail, partial nail avulsion, partial nail avulsion, phenolization, recurrence, wound infection

## Abstract

Background: Ingrown toenails, a common and often painful condition, frequently require surgical intervention for effective management. This study aimed to compare the clinical outcomes of partial nail avulsion (PNA) with and without adjunctive phenolization, specifically focusing on recurrence and postoperative wound infection rates.

Methodology: This prospective comparative interventional study was conducted in the Department of General Surgery at Jinnah Hospital, Lahore, over a six-month period from July 15, 2023, to January 14, 2024. A total of 140 patients were included and randomly divided into two groups: 70 patients in Group A (PNA with phenolization) and 70 patients in Group B (PNA without phenolization). For categorical variables, statistical analysis was conducted using the chi-square test; a p-value of 0.05 was regarded statistically significant. To assess other influencing elements, a subgroup study by age and gender was carried out.

Results: Recurrence was significantly lower in Group A with one patient (1.43%) compared to seven patients (10.0%) in Group B (p=0.029). Wound infection was also reduced in Group A, occurring in four patients (5.71%) versus 12 patients (17.14%) in Group B (p=0.034). Among younger patients (12-35 years), recurrence was 0 out of 46 (0.0%) in Group A versus 6 out of 41 (14.63%) in Group B (p=0.007), while in older patients (36-60 years), recurrence rates were similar (4.17% vs. 3.45%; p=0.891). Female patients in Group A had no recurrence (0 out of 31; 0.0%) compared to 4 out of 32 (12.5%) in Group B (p=0.042).

Conclusion: Our findings indicate that PNA combined with phenolization offers superior outcomes in reducing recurrence rates, especially among younger and female patients, compared to PNA alone. While overall infection rates were lower with phenolization, stratified analysis did not consistently show statistical significance, and no notable adverse effects on wound healing were observed, supporting phenol's clinical utility with minor limitations.

## Introduction

A common podiatric disorder known as onychocryptosis, or ingrown toenails, results from a nail edge growing into the periungual skin, causing inflammation, discomfort, and infection [1]. Usually affecting the great toe, this disorder may greatly reduce a patient's quality of life by limiting everyday activities and mobility [2]. Ingrown toenails arise from many predisposing causes, including trauma, anatomical variances, poor nail cutting, tight shoes, and hereditary tendency. The disorder is divided into many phases ranging from moderate inflammation to chronic infection and granulation tissue development [3,4].

Severity affects management techniques for ingrown toenails. Early on, conservative therapies like warm soaks, appropriate nail cutting methods, and topical or systemic antibiotics are very successful [5]. Surgical intervention becomes essential, nevertheless, in situations of ongoing discomfort, infection, or repeated episodes [6]. Among the many surgical methods accessible, partial nail avulsion (PNA) is a commonly used one involving the removal of the afflicted section of the nail [7]. PNA offers symptomatic alleviation, but recurrence is still a problem, and additional treatments are thus necessary to stop nail regrowth in the impacted region [8].

One such supplementary method is phenolization of the nail matrix, wherein 88% phenol is sprayed on the exposed nail bed after PNA to accomplish chemical matricectomy [9]. A caustic chemical, phenol efficiently damages the nail matrix, therefore lowering the possibility of regeneration and recurrence [10]. Though effective, questions about possible complications, including delayed wound healing, post-procedural discomfort, and localized tissue damage, exist [11,12]. Variations in success rates and complication profiles between PNA alone and PNA with phenolization have been recorded in studies, so further comparative studies are necessary to identify the best treatment plan [5].

Given the clinical relevance and recurrence risk linked with ingrown toenails, it is essential to assess the efficacy of many surgical techniques. This research is to provide a comparison of PNA alone to PNA with phenolization in order to guide doctors in choosing the most efficient strategy for long-term control.

Objective

This study aimed to evaluate and compare the rates of recurrence and postoperative wound infection in patients with Stage 2 and Stage 3 ingrown toenails undergoing PNA, with or without adjunctive phenolization.

## Materials and methods

Study design and setting

This prospective observational study was conducted at the Department of General Surgery, Jinnah Hospital, Lahore, from 15th July 2023 to 14th January 2024.

Sample size calculation

A total sample size of 140 patients (n=70 per group) was determined based on the study by Al-Subaihi [13]. This calculation considered an 80% study power, a 5% (0.05) level of significance [13], and an equal allocation ratio (1:1) between the two study groups. The expected postoperative infection rates used for this calculation were 11% for Group A (partial nail avulsion (PNA) with phenolization) and 0.9% for Group B (PNA without phenol). These rates were derived from existing literature; specifically, a randomized controlled trial by Khan et al. [14] indicated a lower postoperative infection rate in a phenolization group, supporting the 11% assumption for Group A. Furthermore, a retrospective cohort study by Modha et al. [15], which reported an overall postoperative infection rate of 3% in patients undergoing partial or total nail avulsion, also supported the expectation of a lower infection rate in the absence of phenolization for Group B, despite their reported rate being slightly higher than the 0.9% assumed.

Sample technique

Patients were recruited into the study using a non-probability, sequential sampling method.

Participant selection criteria

Inclusion Criteria

Participants eligible for the study were between 12 and 60 years of age, of either gender, and diagnosed with Stage 2 or Stage 3 ingrown toenails. Stage 2 of the condition is characterized by increased pain, swelling, and the presence of pus or drainage, indicating infection. Stage 3 involves further inflammation, the formation of granulation tissue, and hypertrophy of the surrounding nail fold tissues.

Exclusion Criteria

Patients were excluded if they presented with malignant illness, Hepatitis B or C infection, or HIV infection. Additionally, individuals with diabetes mellitus having uncontrolled blood glucose levels (blood sugar random (BSR) >200 mg/dl), peripheral vascular disease, onychomycosis, or those currently undergoing anticoagulant treatment were not included in the study.

Study procedures and data collection

Enrollment and Baseline Data Collection

Following ethical approval from the hospital’s review board, 140 eligible patients were enrolled from the surgical outpatient department. Informed consent was obtained from all participants. Demographic and clinical data were documented, including name, gender, body mass index (BMI), duration of the ingrown toenail condition, and contact information.

Randomization and Interventions

Patients were randomized into two groups (Group A and Group B) using the lottery method. All procedures were performed in a day-care setting under a 2% xylocaine ring block for local anesthesia. For patients in Group A (PNA with phenolization), after PNA, 88% phenol was applied to the exposed nail matrix for a total duration of three minutes using a sterile cotton-tipped applicator. The phenol was reapplied every 30 seconds to ensure consistent chemical contact, and protective petroleum jelly was applied to the surrounding skin to prevent chemical burns. Patients in Group B (PNA without phenolization) underwent PNA only, with no subsequent phenol application.

Post-Procedure Care and Education

All patients received standardized wound care instructions upon discharge on the same day. Comprehensive post-procedure education was provided to both groups, focusing on preventive measures to reduce recurrence risk. This education included guidance on proper toenail trimming techniques, such as cutting nails straight across and avoiding rounded corners, the importance of maintaining good foot hygiene, and the necessity of avoiding tight or ill-fitting footwear.

Follow-Up and Outcome Assessment

Follow-up evaluations for wound infection were conducted on postoperative days 2, 7, and 14. Assessments for the recurrence of ingrown toenails were scheduled at the third month and sixth month post-procedure. To ensure consistency and minimize observational bias, a single, trained observer conducted all follow-up evaluations. Data were systematically recorded using a standardized proforma.

Definition and Monitoring of Key Outcomes

Wound infection was defined by the presence of at least two specific signs at the surgical site: persistent erythema extending beyond the phenolized zone, purulent discharge, local warmth, swelling, or tenderness requiring initiation of systemic antibiotics; minor localized inflammation or mild erythema confined to the phenol-treated zone without systemic signs was not classified as infection. Delayed wound healing was defined as the failure of epithelial closure by postoperative day 21 without signs of infection, and this was monitored through weekly outpatient follow-ups over a four-week period. Patients were also followed up on Day 3, Day 10, and Week 4 to monitor for a range of adverse effects, including wound infection, delayed healing, persistent postoperative pain, phenol-related local tissue damage, allergic reactions, and nail deformity, with standardized case report forms used for documenting any complications. The diagnosis of ingrown toenail was made clinically based on the staging system described in the inclusion criteria (Stage 2 and Stage 3), and these diagnostic criteria guided both participant inclusion and subsequent outcome monitoring.

Statistical analysis

Data analysis was performed using IBM SPSS Statistics for Windows, Version 25 (Released 2017; IBM Corp., Armonk, New York, United States). The normality of continuous data was assessed using the Shapiro-Wilk test. For continuous variables such as age, BMI, and duration of the condition, means and standard deviations (SD) were calculated. For categorical variables like gender and outcomes such as recurrence and infection, frequencies and percentages were computed. The chi-square test was used to compare recurrence and infection rates between Group A and Group B. A p-value ≤0.05 was considered statistically significant. Data were further stratified by age, gender, duration of the condition, and BMI, and post-stratification analysis using the chi-square test was performed to evaluate significance within each subgroup.

Ethical approval

Ethical approval for this study was obtained from the Allama Iqbal Medical College/Jinnah Hospital, Lahore ethical review board (Approval No. 213/10/3/2022/S1 ERB) prior to its commencement. Written informed consent was secured from all participants. Confidentiality of patient data was maintained, and the research was conducted in adherence to established ethical research standards.

## Results

Table 1 shows the baseline characteristics of all 140 patients, equally divided into Group A (n=70) and Group B (n=70). In Group A, 46 patients (65.71%) were aged 12-35 years and 24 patients (34.29%) were aged 36-60 years; similarly, in Group B, 41 patients (58.57%) were aged 12-35 years and 29 patients (41.43%) were aged 36-60 years. The mean age was 27.58 ± 6.56 years in Group A and 28.09 ± 6.66 years in Group B. Gender distribution was similar, with 39 male patients (55.71%) and 31 female patients (44.29%) in Group A, and 38 male patients (54.29%) and 32 female patients (45.71%) in Group B. The duration of disease was ≤6 months in 47 patients (67.14%) in Group A and 46 patients (65.71%) in Group B, while >6 months in 23 patients (32.86%) and 24 patients (34.29%), respectively. BMI ≤25 kg/m² was seen in 37 patients (52.86%) in Group A and 38 patients (54.29%) in Group B, while BMI >25 kg/m² was recorded in 33 patients (47.14%) in Group A and 32 patients (45.71%) in Group B. Mean BMI was comparable at 27.58 ± 6.56 kg/m² in Group A and 28.09 ± 6.66 kg/m² in Group B.

**Table 1:** Baseline characteristics of patients in both groups (n=140). Group A: Partial nail avulsion (PNA) with phenolization; Group B: PNA without phenolization.

Category		Group A (n=70)	Group B (n=70)
Age distribution	12-35 years	46 (65.71)	41 (58.57)
	36-60 years	24 (34.29)	29 (41.43)
	Mean ± SD	27.58 ± 6.56	28.09 ± 6.66
Gender distribution	Male	39 (55.71)	38 (54.29)
	Female	31 (44.29)	32 (45.71)
Duration of disease	≤6 months	47 (67.14)	46 (65.71)
	>6 months	23 (32.86)	24 (34.29)
BMI (kg/m²)	≤25	37 (52.86)	38 (54.29)
	>25	33 (47.14)	32 (45.71)
	Mean ± SD	27.58 ± 6.56	28.09 ± 6.66

Table 2 shows the primary outcomes. Wound infection occurred in four patients (5.71%) in Group A compared to 12 patients (17.14%) in Group B (p=0.034). Recurrence was significantly lower in Group A, with only one patient (1.43%) affected, versus seven patients (10.0%) in Group B (p=0.029).

**Table 2:** Comparison of the outcome of PNA alone versus PNA with phenolization of the nail matrix (n=140). Group A: Partial nail avulsion (PNA) with phenolization; Group B: PNA without phenolization.

Complications	Group A (n=70) Yes (%)	Group A No (%)	Group B (n=70) Yes (%)	Group B No (%)	p-value	χ² value
Wound infection	4 (5.71%)	66 (94.29%)	12 (17.14%)	58 (82.86%)	0.034	4.49
Recurrence rate	1 (1.43%)	69 (98.57%)	7 (10.0%)	63 (90.0%)	0.029	4.76

As shown in Table 3, age-based stratification revealed that among younger patients (12-35 years), wound infection occurred in 3 out of 46 patients (6.52%) in Group A and 6 out of 41 patients (14.63%) in Group B (p=0.215), indicating no statistically significant difference. Among older patients (36-60 years), infection was observed in 1 out of 24 patients (4.17%) in Group A and 6 out of 29 patients (20.69%) in Group B (p=0.077), which was also not statistically significant. These results suggest that the incidence of wound infection did not significantly vary between groups across both age strata.

**Table 3:** Stratification of wound infection and recurrence rate according to age groups in both groups (n=140). Group A: Partial nail avulsion (PNA) with phenolization; Group B: PNA without phenolization.

Complication	Age Group	Group A Yes (%)	Group A No (%)	Group B Yes (%)	Group B No (%)	p-value	χ² value
Wound infection	12–35	3 (6.52%)	43 (93.48%)	6 (14.63%)	35 (85.37%)	0.215	1.54
	36–60	1 (4.17%)	23 (95.83%)	6 (20.69%)	23 (79.31%)	0.077	3.14
Recurrence	12–35	0 (0.0%)	46 (100.0%)	6 (14.63%)	35 (85.37%)	0.007	7.28
	36–60	1 (4.17%)	23 (95.83%)	1 (3.45%)	28 (96.55%)	0.891	0.02

Regarding recurrence, it was significantly lower in younger patients of Group A, where 0 out of 46 patients (0.0%) experienced recurrence compared to 6 out of 41 patients (14.63%) in Group B (p=0.007). However, in the older age group, recurrence was comparable between the two interventions, reported in 1 out of 24 patients (4.17%) in Group A and 1 out of 29 patients (3.45%) in Group B (p=0.891), showing no significant difference.

Table 4 presents the gender-wise analysis. Among male patients, wound infection was observed in 2 out of 39 (5.13%) in Group A and 6 out of 38 (15.79%) in Group B, which was not statistically significant (p=0.125). Among female patients, infection was found in 2 out of 31 (6.45%) in Group A and 6 out of 32 (18.75%) in Group B, also not statistically significant (p=0.143). Regarding recurrence, male patients showed no significant difference: 1 out of 39 (2.56%) in Group A versus 3 out of 38 (7.89%) in Group B (p=0.292). However, in female patients, recurrence was significantly lower in Group A with 0 out of 31 (0.0%) affected, compared to 4 out of 32 (12.5%) in Group B (p=0.042).

**Table 4:** Stratification of wound infection and recurrence rate according to gender in both groups (n=140). Group A: Partial nail avulsion (PNA) with phenolization; Group B: PNA without phenolization.

Complication	Gender	Group A Yes (%)	Group A No (%)	Group B Yes (%)	Group B No (%)	p-value	χ² value
Wound infection	Male	2 (5.13%)	37 (94.87%)	6 (15.79%)	32 (84.21%)	0.125	2.36
	Female	2 (6.45%)	29 (93.55%)	6 (18.75%)	26 (81.25%)	0.143	2.14
Recurrence	Male	1 (2.56%)	38 (97.44%)	3 (7.89%)	35 (92.11%)	0.292	1.11
	Female	0 (0.0%)	31 (100.0%)	4 (12.50%)	28 (87.50%)	0.042	4.12

As shown in Table 5, duration-based stratification indicated higher wound infection in Group B for both short and long disease durations. For patients with disease duration ≤6 months, wound infection occurred in 3 out of 47 (6.38%) in Group A and 9 out of 46 (19.57%) in Group B (p=0.058). Among those with >6 months of disease, infection was seen in 1 out of 23 (4.35%) in Group A versus 3 out of 24 (12.50%) in Group B (p=0.317). Recurrence in patients with ≤6 months of disease was 1 out of 47 (2.13%) in Group A and 5 out of 46 (10.87%) in Group B (p=0.086). For disease >6 months, recurrence was seen in 0 out of 23 (0.0%) in Group A and 2 out of 24 (8.33%) in Group B (p=0.157). The mean healing time was 13.2 ± 2.1 days in Group A and 12.9 ± 2.3 days in Group B. The difference was not statistically significant (p = 0.421), suggesting that adjunctive phenolization does not adversely affect the postoperative recovery timeline. Delayed wound healing occurred in two patients (2.86%) in Group A and three patients (4.29%) in Group B, with no statistically significant difference (p = 0.648). These cases resolved spontaneously without secondary intervention.

**Table 5:** Stratification of wound infection and recurrence rate according to the duration in both groups (n=140). Group A: Partial nail avulsion (PNA) with phenolization; Group B: PNA without phenolization.

Complication	Duration	Group A Yes (%)	Group A No (%)	Group B Yes (%)	Group B No (%)	p-value	χ² value
Wound infection	≤6 months	3 (6.38%)	44 (93.62%)	9 (19.57%)	37 (80.43%)	0.058	3.61
	>6 months	1 (4.35%)	22 (95.65%)	3 (12.50%)	21 (87.50%)	0.317	1.00
Recurrence	≤6 months	1 (2.13%)	46 (97.87%)	5 (10.87%)	41 (89.13%)	0.086	2.96
	>6 months	0 (0.0%)	23 (100.0%)	2 (8.33%)	22 (91.67%)	0.157	2.00

Table 6 shows BMI-based outcomes. For patients with BMI ≤25 kg/m², wound infection was observed in 2 out of 37 (5.41%) in Group A and 7 out of 38 (18.42%) in Group B (p=0.083). In those with BMI >25 kg/m², infection occurred in 2 out of 33 (6.06%) in Group A versus 5 out of 32 (15.63%) in Group B (p=0.214). Recurrence for BMI ≤25 kg/m² was reported in 1 out of 37 (2.70%) in Group A compared to 6 out of 38 (15.79%) in Group B (p=0.051). For BMI >25 kg/m², recurrence was seen in 0 out of 33 (0.0%) in Group A and 1 out of 32 (3.23%) in Group B (p=0.306).

**Table 6:** Stratification of wound infection and recurrence rate according to BMI in both groups (n=140). Group A: Partial nail avulsion (PNA) with phenolization; Group B: PNA without phenolization.

Complication	BMI Category	Group A Yes (%)	Group A No (%)	Group B Yes (%)	Group B No (%)	p-value	χ² value
Wound infection	≤25	2 (5.41%)	35 (94.59%)	7 (18.42%)	31 (81.58%)	0.083	3.03
	>25	2 (6.06%)	31 (93.94%)	5 (15.63%)	27 (84.37%)	0.214	1.54
Recurrence	≤25	1 (2.70%)	36 (97.30%)	6 (15.79%)	32 (84.21%)	0.051	3.82
	>25	0 (0.0%)	33 (100.0%)	1 (3.23%)	31 (96.77%)	0.306	1.04

## Discussion

The findings of this study demonstrate that PNA combined with phenolization leads to a statistically significant reduction in both recurrence and wound infection rates in the treatment of ingrown toenails compared to PNA alone. These results underscore the clinical benefits of chemical matricectomy using phenol, aligning with and building upon previous research.

Enhanced efficacy with phenolization: recurrence and infection rates

A primary outcome of this research was the marked difference in recurrence rates. The PNA with phenolization group (Group A) exhibited a recurrence rate of 1.43%, significantly lower than the 10.0% observed in the PNA alone group (Group B) (p=0.029). This supports the efficacy of phenol in achieving chemical matricectomy. Similarly, wound infection rates were substantially lower in Group A (5.71%) compared to Group B (17.14%) (p=0.034). These observations are consistent with earlier investigations by Bos et al. [16] and Khan et al. [14], which also highlighted the effectiveness of phenol. For instance, research by Bos et al. [16] reported a recurrence rate of 4.2% for PNA with phenolization versus 29.6% for PNA alone, emphasizing the superior long-term outcomes of phenol treatment in preventing nail regrowth. Our data corroborate these findings, reinforcing the role of phenol in reducing recurrence.

Regarding wound infection, the significantly lower incidence in the phenolization group aligns with other studies that have reported similar benefits, such as infection rates of 4.9% with phenolization versus 18.3% without [17,18]. The antiseptic properties of phenol, which contribute to sanitizing the nail bed and preventing bacterial colonization, likely explain its utility in reducing postoperative infections.

Subgroup analyses: age and gender-specific benefits

Our study further indicates that younger patients (12-35 years) with ingrown toenails derive considerable benefit from phenolization following PNA. Within this age demographic, Group A experienced no recurrences (0.0%), while Group B had a recurrence rate of 14.63% (p=0.007). This finding is consistent with previous research suggesting that younger individuals, potentially due to more active nail matrix activity, may face higher recurrence rates if phenolization is not employed [17,18].

Gender-based analysis also revealed a significant advantage for phenolization among female participants, where the recurrence rate was 0.0% in the phenolization group compared to 12.5% in the PNA alone group (p=0.042). This aligns with studies suggesting women may experience superior cosmetic outcomes and reduced recurrence rates post-phenolization [19]. However, it is noteworthy that while phenolization significantly reduced recurrence in female patients, no statistically significant difference was observed in wound infection rates between the treatment groups within this specific subgroup (p>0.05). This suggests that the antiseptic effects of phenol might not manifest as distinctly in gender-stratified infection analyses, possibly due to factors such as lower baseline infection rates or sample size limitations within the female cohort.

Wound healing and procedural safety

Despite some historical concerns in the literature that phenolization might delay wound healing, our study did not observe any significant increase in healing time-related complications. Both treatment groups exhibited similar recovery trajectories, and no severe adverse events were reported. This finding is congruent with studies such as that by Silva et al. [20], who also reported no significant differences in the wound healing duration between phenolization and other surgical techniques. While our study did find a lower overall wound infection rate in the phenol group (5.71% vs. 17.14%, p = 0.034), the subgroup stratification by age and gender did not yield statistically significant differences in infection rates across all strata. This may limit the generalizability of the infection-related benefit to specific demographics based on our current data. Nevertheless, the absence of delayed healing or severe adverse effects in either group supports the procedural safety of phenolization. Given the low incidence of delayed healing in both groups, our study may have been underpowered to detect minor differences in this specific outcome.

Considerations on the surgical technique

In our protocol, PNA was performed without complete surgical excision of the nail bed. Instead, chemical matricectomy using 88% phenol was employed to selectively ablate the germinal matrix while preserving surrounding soft tissue. This technique aligns with the method described by Bos et al. [16], who demonstrated effective matrix destruction with minimal collateral tissue damage using phenol. Mechanical curettage or surgical excision of the nail bed was intentionally avoided to minimize postoperative morbidity and preserve the nail’s cosmetic appearance. While some surgical approaches advocate for direct excision or curettage of the nail matrix, particularly in recurrent cases, multiple comparative studies [14,16,19] have indicated that phenolization alone, when applied correctly and for an adequate duration, is sufficient for effective matricectomy. Furthermore, by avoiding surgical removal of the nail bed, the risks of excessive granulation tissue formation, prolonged healing time, and cosmetic deformity are reduced, factors particularly relevant for younger or cosmetically concerned patients.

Interpretation of subgroup infection rates

Although subgroup analyses for age and gender did not consistently demonstrate statistically significant differences in wound infection rates, the overall reduction in infection observed in the phenolization group (Group A) was statistically significant. The lack of statistical significance in certain subgroups may be attributable to limited sample sizes within those strata rather than a true absence of clinical benefit. The observed trends, generally lower infection rates in most phenol subgroups, are consistent with phenol’s established antiseptic properties and support its continued use as a valuable adjunct in ingrown toenail surgery.

Study strengths and limitations

The prospective design of this study and its direct comparison of PNA with and without phenolization represent key strengths, providing robust evidence to inform clinical decision-making. Additionally, the stratification of recurrence and infection rates by age and gender offers insightful analysis of subgroup-specific outcomes.

However, certain limitations should be acknowledged. The relatively small sample size may affect the generalizability of the findings. Furthermore, as the research was conducted at a single center, this could limit its external validity. While a six-month follow-up duration was chosen, we recognize that late recurrences, particularly in cases of phenol matricectomy failure, can occasionally present beyond this timeframe. Future studies with larger, multi-center cohorts and potentially longer follow-up periods would be beneficial to further validate these findings and explore the nuances within specific demographic subgroups.

## Conclusions

Our research shows that, in the therapy of ingrown toenails, PNA plus phenolization is a better strategy than PNA alone in greatly lowering recurrence and infection rates. The results support phenol's effectiveness in stopping post-procedural problems and nail regrowth. Although there are some small issues with delayed wound healing, generally phenolization is a better treatment choice for ingrown toenails as its advantages exceed its hazards.

PNA combined with phenolization is associated with a significantly lower recurrence rate compared to PNA alone, particularly among younger and female patients. While wound infection rates were lower in the phenol group overall, subgroup analysis did not reveal statistically significant differences in all strata. Additionally, no adverse effects on healing were observed in the phenolization group, though these findings should be interpreted with caution due to sample size limitations in stratified subgroups.
